# Emergency Physicians’ Familiarity with the Safe Handling of Firearms

**DOI:** 10.5811/westjem.2018.11.39822

**Published:** 2018-11-30

**Authors:** Andrew R. Ketterer, Kaitlin Ray, Anne Grossestreuer, Nicole Dubosh, Edward Ullman, Matthew Pirotte

**Affiliations:** * Beth Israel Deaconess Medical Center, Department of Emergency Medicine, Boston, Massachusetts; † Northwestern University McGaw Medical Center, Department of Emergency Medicine, Chicago, Illinois

## Abstract

**Introduction:**

Emergency physicians (EP) experience high rates of workplace violence, the risks of which increase with the presence of weapons. Up to 25% of trauma patients brought to the emergency department (ED) have been found to carry weapons. Given these risks, we conducted an educational needs assessment to characterize EPs’ knowledge of firearms, frequency of encountering firearms in the ED, and level of confidence with safely removing firearms from patient care settings.

**Methods:**

This was a survey study of attending and resident EPs at two academic and four community hospitals in the Midwest and Northeast. A 26-item questionnaire was emailed to all EPs at the six institutions. Questions pertained to EPs’ knowledge of firearms, experience with handling firearms, and exposure to firearms while at work. We calculated response proportions and p-values.

**Results:**

Of 243 recipients who received the survey, 149 (61.3%) completed it. Thirty-three respondents (22.0%) reported encountering firearms in the workplace, 91 (60.7%) reported never handling firearms, and 25 (16.7%) reported handling firearms at least once per year. Thirty-six respondents (24.0%) reported formal firearms training, and 63 (42.3%) reported no firearms training. There were no significant regional differences regarding firearms training or exposure. Residents from the Northeast were more likely to be moderately confident that they could safely handle a firearm prior to law enforcement involvement (p=0.043), while residents from the Midwest were more likely to be not at all confident (p=0.018).

**Conclusion:**

The majority of surveyed attending and resident EPs reported little experience with handling firearms. Among resident EPs, there was a regional difference in confidence in handling firearms prior to law enforcement involvement. Given the realities of workplace violence and the frequency with which firearms are encountered in the ED, further investigation is needed to evaluate provider competence in safely handling them. EPs may benefit from training on this topic.

## INTRODUCTION

Violence in the emergency department (ED) is a well-known occurrence, with 75% of emergency physicians (EP) experiencing at least one violent incident in the workplace every year.^1^ Of particular concern in this context is the possibility for the introduction of weapons into the ED. Aspects of EDs designed to improve patients’ access to care, specifically open walk-in entry areas and waiting rooms, inadvertently allow for easier entry of weapons.^2^ One study estimates that 20% of EDs in the United States have guns or knives brought in on a daily or weekly basis.^3^ While the majority of ED workplace violence consists of verbal threats and physical assaults without the use of weapons,^4^ the potential threat of firearms in particular is of ongoing concern.

Analysis of hospital-based shootings reveals that one third occur in the ED or in the immediate surrounding areas (ambulance ramp, ED parking lot, waiting room).^5^ EDs in southern states, hospitals with more than 400 beds, EDs seeing more than 60,000 patients per year, and Level I trauma centers are at particular risk.^4^–^6^ One retrospective study found that 26% of major trauma patients were armed with lethal weapons, and guns comprised 16.3% of the weapons confiscated from these patients.^6^ Moreover, guns brought into the ED by patients represent only a part of the problem; among safety incidents involving firearms, 50% involved a security personnel member’s firearm. These findings suggest that the presence of any firearm in this high-stress environment can be a threat to patient and staff safety.^5^

Clearly, EPs are at risk for exposure to guns while at work in the ED. While we are not aware of any published data on the likelihood that EPs will be required to handle firearms at work (e.g., removing a firearm from a patient’s person or belongings during a trauma assessment), the risk for such an event is concerning. There is also a paucity of published data on accidental firearms discharges in the ED, however these events represent a real risk for injury. Epidemiologic data from the community show that a large number of injuries due to accidental firearms discharge result from routine activities such as carrying, showing, or looking at a gun, with one study estimating the incidence of these mechanisms at 23.9%.^7^ A more recent study showed that 35.3% of patients presenting to the ED with firearm injuries sustained unintentional injuries.^8^ These findings highlight the risks associated with merely handling firearms, particularly among those inexperienced with doing so.^9^ To our knowledge, no studies have specifically assessed the exposure of EPs to firearms or EPs’ confidence in handling them. Given the unpredictable nature of the ED and the potential for entry of firearms, it is evident that data is lacking regarding the risks of EP encounters with firearms in the workplace.

The purpose of this study was to determine the frequency with which EPs encounter firearms while at work in the ED, characterize EPs’ experience with handling firearms, and describe EPs’ level of confidence with safely handling a firearm should one be encountered in the ED.

## METHODS

### Study Design

This was a survey study of resident and attending EPs at two academic and four community hospitals in the Midwest and Northeast. Our survey tool was developed using an iterative process in keeping with published best practices in survey design.^10^,^11^ We conducted a literature review to identify relevant variables in EPs’ exposure to firearms. After developing survey items in keeping with the terminology and data present in the relevant literature, we assessed for content validity of the survey items using local content expert review. Content experts included academic emergency medicine (EM) faculty with experience in survey design methodology and EM faculty with training in firearms handling, defined as having undergone a formal gun safety course such as concealed-carry training, tactical firearms training, etc. Experts reviewed the wording of each item for clarity, content, and utility, and their comments were integrated into the survey. After assessment for content validity the survey was administered to EM faculty to assess for response process validity using immediate retrospective probing.^10^ Their impressions of each item were recorded and integrated into the final version of the survey.^12^ After finalizing survey items, the survey was electronically delivered to the study population.

**Population Health Research Capsule**

What do we already know about this issue?

*The emergency department is at risk for the entry of guns. Guns represent a safety risk for patients and staff. A large proportion of gun injuries are due to accidental discharges during handling*.

What was the research question?

How often do emergency physicians (EPs) encounter guns? What experience and level of confidence do EPs have with handling guns?

What was the major finding of the study?

*The surveyed EPs report encountering guns at a low but measurable rate. Respondents have little experience with handling guns*.

How does this improve population health?

*Our findings demonstrate a knowledge gap among the surveyed EPs that has implications for workplace safety. EPs may benefit from training on the topic of firearms safety*.

### Study Population

The study population included resident and attending EPs at two academic hospitals and their community affiliate hospitals in the Midwest and Northeast. As EDs have been found to be at higher risk for firearms encounters,^5^ these two populations represent the most likely physicians to be exposed to firearms in the hospital setting.

### Study Protocol

A questionnaire was emailed to EPs whose primary clinical duties were at the included institutions (Appendix). All survey responses were anonymous. A total of three reminders were sent to all respondents. We conducted the survey questionnaire using Google Forms, and stored all data in a password-protected online file. This study was considered exempt by the institutional review board of Northwestern University.

### Key Outcome Measures

We sought to characterize multiple facets of EPs’ exposure, confidence, and experience with handling firearms, and the frequency with which they encounter firearms while on duty in the ED.

### Data Analysis

We analyzed survey results using Stata (14.2). Response rates were calculated using the calculator tool provided by the American Association for Public Opinion Research.^13^ We calculated response proportions for each question, and p values were calculated using chi^2^ and Fisher’s exact test.

## RESULTS

Of 243 recipients who were sent the survey, 149 (61.3%) completed it. Demographic data of respondents can be found in Table 1. Respondents from the Midwest included 40 of 58 resident EPs (70.0%) and 21 of 44 attending EPs (47.7%), while respondents from the Northeast included 24 of 36 resident EPs (66.7%) and 64 of 115 attending EPs (55.7%). There were no significant regional differences in response rates of attending or resident EPs, nor were there significant regional differences in response rates of men vs. women.

Twenty-five percent of resident EPs and 20% of attending EPs reported encountering firearms in the ED or its immediate environment. Of these, few respondents reported encountering firearms in the workplace on a daily, weekly, or monthly basis, with the majority reporting encountering firearms on a yearly or less-often basis. We observed no significant differences in level of training or geographic region regarding rates of firearms exposure in the workplace (Table 2).

Personal experience with handling firearms was similarly low, with 90 respondents (60.4%) reporting never handling a firearm in their daily lives. Attending EPs were significantly more likely than resident EPs to report never handling firearms in their daily lives (p=0.003), with no significant regional differences found within either group. Of those who reported handling firearms, there was a trend toward resident EPs being more likely than attending EPs to report having undergone formal or informal firearms training (p=0.06). Attending EPs were significantly more likely than resident EPs to report having no firearms training (p=0.018). No significant regional differences in firearms training were found among either resident or attending EPs (Table 3).

Confidence in handling a firearm found in a patient’s possession until it could be safely turned over to law enforcement was varied, but each confidence level was fairly evenly distributed when comparing resident EPs to attendings (Table 4). No significant differences in level of confidence were found between resident and attending EPs, nor were there significant regional differences among attending EPs. Resident EPs from the Northeast were significantly more likely to be “moderately” confident that they could safely handle a firearm found in a patient’s possession (p=0.043), while resident EPs from the Midwest were significantly more likely to be “not at all” confident that they could do so (p=0.018). Attending EPs were significantly more likely than resident EPs to report knowing whether their hospital had a protocol regarding the handling and management of firearms found in a patient’s possession, while residents were significantly more likely to be unsure whether their hospital had a protocol (p=0.001) (Table 5). No significant regional differences were found regarding knowledge of such hospital protocols.

## DISCUSSION

We found that the majority of EPs at the surveyed institutions reported little experience with safely handling firearms. At the same time, a cumulative 20% of responding attendings and 25% of responding residents reported encountering firearms while at work in the ED. In some ways this “low frequency high risk” encounter is analogous to other unique scenarios in EM such as performing an ED thoracotomy or peri-mortem caesarean section. These rare but crucial procedures receive high levels of educational attention, as EPs must be able to perform them in the event they are needed. The majority of our respondents reported little or no experience with handling firearms, showing a knowledge gap. Further investigation is needed to assess the prevalence of this knowledge gap among resident and attending EPs generally. A national knowledge gap in this area would suggest a general need for firearms education that may have implications for workplace safety, as accidental firearms discharge in the ED should be considered a “never event.” Although such education may not translate to confidence in handling firearms, just as with other “low frequency high risk” procedures, EPs may benefit from subject matter familiarity in the event that they are required to remove a firearm from the clinical care environment.

The surveyed population had heterogeneous levels of experience with handling firearms, with resident EPs being more likely to have handled firearms in their daily lives. This coincides with the fact that the surveyed residents were more likely to have had formal or informal firearms training than the surveyed attendings. The survey was not calibrated to investigate the nature of this training; for example, it is possible that a higher percentage of the surveyed residents served in the military. Nevertheless, this difference suggests variability among EPs in firearms training and personal familiarity with firearms. Further investigation may be needed to assess the generalizability of these findings and could help elucidate the exact education and exposures that lead to the intergroup differences we found in this study.

Resident respondents in the Northeast were significantly more likely than those in the Midwest to report confidence that they could safely handle firearms encountered in the clinical environment. The driver for this difference is unclear, as very few other regional differences existed in the surveyed populations. The likelihood of encountering firearms in the clinical environment was not significantly different between respondents from the Midwest and respondents from the Northeast. Similarly, these groups were not significantly different in the extent to which they have received firearms training or the frequency with which they handle firearms in their daily lives. Comparison of resident respondents in the Midwest vs. the Northeast and attending respondents in the Midwest vs. the Northeast also yielded no significant differences. A performance-based needs assessment could help evaluate the actual baseline level of ability residents have in safely handling firearms found in the clinical environment.

Finally, attending respondents were significantly more likely than resident respondents to report knowledge of hospital protocols regarding the handling and management of firearms found in patient possession. This may be driven by the greater involvement of attending EPs with departmental and hospital administration, leading to greater familiarity with hospital protocols in general. However, despite their comparatively greater familiarity with the presence or absence of a hospital protocol, the majority of attending respondents were unsure of whether or not their hospital had a protocol regarding patients’ firearms. These findings, therefore, highlight a knowledge gap among both residents and attendings that suggests a need for additional education for workplace safety training. Particularly given that resident and attending EPs may be called upon to remove firearms from the clinical setting, familiarity with hospital protocols surrounding this action may be critical.

## LIMITATIONS

This study has several important limitations. Respondent level of training differed between the Midwest and Northeast, with significantly more residents than attendings responding from the Midwest and vice versa from the Northeast (p<0.001). This makes interpretation of these two geographic regions problematic, as the average level of training differs between these two respondent populations. This effect is mitigated by analyzing the data within groups defined by resident- and attending-level of training. An additional limitation is the lack of pilot administration during survey design, potentially limiting its internal validity. This was due to the relatively small size of some of our sub-populations (e.g., 36 residents in the Northeast). Administration of a pilot would have rendered those respondents ineligible for analysis in the final survey, as participation in both would potentially have created an exposure bias. Decreasing the number of potential respondents eligible for analysis would effectively decrease our maximum response rate and thereby reduce the study’s power to detect differences between groups. Given this risk and in light of our otherwise-rigorous development of the survey tool, we elected to proceed with the survey in lieu of a pilot study. The similar response rates to all questions except as noted above suggest that our survey tool is very likely to have a high degree of internal validity.

A final limitation is the fact that this study was conducted at only two academic centers and their community affiliates. While our response rate is likely to be representative of the surveyed population,^14^ this population represents only a small portion of the total number of resident and attending EPs in the surveyed regions. Furthermore, some regions not represented in our study are at higher risk for hospital-based firearms violence. These factors greatly limit this study’s generalizability to the country as a whole. A multi-center investigation including hospital systems in the American South, Southeast, and West could help elucidate whether EP experience and confidence in handling firearms is related to the regional variability seen in firearms ownership^15^ and firearms violence.^4^,^5^ With this in mind, geographic differences and differences between levels of training were found in the surveyed populations, which may suggest even greater heterogeneity among EPs nationally. Further investigation may be needed to better characterize the degree of variability among EPs.

## CONCLUSION

The majority of EPs at the surveyed institutions report little experience with handling firearms. While our survey shows that firearms are infrequently encountered in the clinical environment, a low level of exposure is nevertheless apparent among our surveyed population. Given the high risks associated with handling firearms and the fact that accidental firearms could discharge during removal from the patient bedside should be considered a “never event,” it may be beneficial for EPs to receive training in safely handling firearms. Finally, respondents were largely unaware of the presence or absence of protocols at their home institutions regarding handling of firearms found in a patient’s possession. EPs may benefit from dedicated training on these topics.

## Supplementary Material



## Figures and Tables

**Table 1:** Demographics of emergency physicians who responded to survey regarding familiarity with handling firearms.

	Midwest (n=61)	Northeast (n=88)
Attending [n(%)]	21 (34.4)	64 (72.3)
Resident [n(%)]	40 (65.6)	24 (27.3)
Male [n(%)]	41 (67.2)	62 (70.5)
Practice site – Urban [n(%)]	59 (96.7)	76 (86.4)
Practice site – Suburban [n(%)]	2 (3.3)	10 (11.4)
Practice site – Rural [n(%)]	0 (0.0)	2 (2.3)
Practice site – Level 1 Trauma Center [n(%)]	59 (96.7)	57 (64.8)
Practice site – Trauma Center, not Level 1 [n(%)]	2 (3.3)	2 (2.3)
Practice site – not a Trauma Center [n(%)]	0 (0.0)	29 (33.0)

**Table 2:** Reported frequency of encountering firearms in the emergency department or its immediate surrounding areas.

How often do you personally encounter firearms in your primary emergency department or its immediate environment (waiting room, parking lot, ambulance bay, etc.)? (n=149)	Attending Midwest (n=21)	Attending Northeast (n=64)	P value	Resident Midwest (n=40)	Resident Northeast (n=24)	P value
Never or blank	18 (86)	50 (78)	0.545	31 (78)	17 (71)	0.565
Less frequently, but I do personally encounter firearms in/around the emergency department	2 (10)	5 (8)	0.805	4 (10)	3 (13)	0.756
Yearly	1 (5)	4 (6)	0.801	3 (8)	2 (8)	0.904
Monthly	0 (0)	1 (2)	0.565	1 (3)	2 (8)	0.285
Weekly	0 (0)	1 (2)	0.565	1 (3)	0 (0)	0.435
Daily	0 (0)	3 (5)	0.312	0 (0)	0 (0)	n/a

**Table 3:** Resident and attending emergency physicians’ reported degree of personal experience with handling firearms.

	Attending (n=85)	Resident (n=64)	P value
How often do you handle firearms in your daily life? (n=149)			
Never	60 (71)	30 (47)	^*^0.003
How often do you personally encounter firearms in your primary emergency department or its immediate environment (waiting room, parking lot, ambulance bay, etc.)? (n=149)			
Never or blank	68 (80)	48 (75)	0.444
To what extent have you had firearms training? (n=149)			
Formal	18 (21)	18 (28)	0.060
Informal	24 (28)	26 (41)	0.060
None	43 (51)	20 (31)	^*^0.018
If you were to encounter a firearm in a patient’s possession, how confidently do you feel you could safely handle it until it can safely be turned in to law enforcement? (n=149)			
Extremely	14 (16)	9 (14)	0.895
Moderately	20 (24)	12 (19)	0.895
Somewhat	10 (12)	9 (14)	0.895
Slightly	18 (21)	17 (27)	0.895
Not at all	23 (27)	17 (27)	0.895

**Table 4:** Resident and attending emergency physicians’ reported degree of confidence in handling firearms encountered in a patient’s possession.

If you were to encounter a firearm in a patient’s possession, how confidently do you feel you could safely handle it until it can safely be turned in to law enforcement? (n=149)	Attending Midwest (n=21)	Attending Northeast (n=64)	P value	Resident Midwest (n=40)	Resident Northeast (n=24)	P value
Extremely	1 (5)	13 (20)	0.172	4 (10)	5 (21)	0.228
Moderately	5 (24)	15 (23)	0.972	4 (10)	8 (33)	^*^0.043
Somewhat	3 (14)	7 (11)	0.679	6 (15)	3 (13)	0.781
Slightly	4 (19)	14 (22)	0.783	11 (28)	6 (25)	0.827
Not at all	8 (38)	15 (23)	0.190	15 (38)	2 (8)	^*^0.018

**Table 5:** Resident and attending emergency physicians’ reported degree of knowledge of hospital protocols regarding management of firearms discovered in patients’ possession.

Are you aware of a hospital protocol regarding handling and management of firearms discovered in the possession of patients within your primary emergency department? (n=149)	Attending (n=85)	Resident (n=64)	P value
Yes it does	29 (34)	6 (9)	^*^0.001
No it doesn't	1 (1)	0 (0)	^*^0.001
Unsure	55 (65)	58 (91)	^*^0.001
