# Supplementary material for: Emergency Physicians’ Familiarity with the Safe Handling of Firearms
Source: West J Emerg Med. 2018 Nov 30;20(1):170–6. doi: 10.5811/westjem.2018.11.39822 (PMC6324699; doi:10.5811/westjem.2018.11.39822)
Supplement: Supplementary file 1 [file wjem-20-170-s001.pdf]

Familiarity of emergency physicians with the safe handling of firearms: A needs assessment survey

1. **Which of the following best describes your role in the Emergency Department?**
Mark only one oval.

- Resident PGY1
- Resident PGY2
- Resident PGY3
- Resident PGY4
- Fellow
- Attending
- Other: ______________________

2. **Please select the state in which you are currently training/practicing:**
Mark only one oval.

- Alabama
- Alaska
- Arizona
- Arkansas
- California
- Colorado
- Connecticut
- Delaware
- Florida
- Georgia
- Hawaii
- Idaho
- Illinois
- Indiana
- Iowa
- Kansas
- Kentucky
- Louisiana
- Maine
- Maryland
- Massachusetts
- Michigan
- Minnesota
- Mississippi
- Missouri
- Montana
- Nebraska
- Nevada
- New Hampshire
- New Jersey
- New Mexico
- New York
- North Carolina
- North Dakota
- Ohio

- Oklahoma
- Oregon
- Pennsylvania
- Rhode Island
- South Carolina
- South Dakota
- Tennessee
- Texas
- Utah
- Vermont
- Virginia
- Washington
- West Virginia
- Wisconsin
- Wyoming

3. **What description best characterizes your primary emergency department?**
Mark only one oval.

- Urban
- Suburban
- Rural

4. **What is the yearly census of your primary emergency department?**
Mark only one oval.

- <40,000 visits/year
- 40,000-59,999 visits/year
- 60,000-79,999 visits/year
- 80,000-100,000 visits/year
- >100,000 visits/year

5. **What is your primary emergency department's status as a trauma center?**
Mark only one oval.

- Level 1
- Trauma center, not level 1
- Not a trauma center

6. **Please select the gender you most identify with:**
Mark only one oval.

- Male
- Female
- Prefer not to say

Firearms exposure

7. **How many times during the week do you feel acutely unsafe with regards to your physical well-being while at work in the emergency department?**
Mark only one oval.

- Never
- 1-2 times per week
- 3-4 times per week
- Daily
- More than once per shift

8. **How often do you handle firearms in your daily life?**
Mark only one oval.

- Daily
- Weekly
- Monthly
- Yearly
- Less frequently, but I do handle firearms
- Never *Skip to question 11.*

Firearms exposure

9. **Which type of firearm have you handled in your daily life? (select all that apply)**
Check all that apply.

- [ ] Handgun, revolver
- [ ] Handgun, semiautomatic
- [ ] Rifle
- [ ] Shotgun
- [ ] Military-style weapon (e.g. AR15, AK-47, SKS)
- [ ] Pellet or airgun
- [ ] I have never encountered a firearm

10. **How often do you personally encounter firearms in your primary emergency department or its immediate environment (waiting room, parking lot, ambulance bay, etc.)?**

Mark only one oval.

- Daily
- Weekly
- Monthly
- Yearly
- Less frequently, but I do personally encounter firearms in/around the ED
- Never

Firearms training

11. **To what extent have you had firearms training?**

Mark only one oval.

- I have had FORMAL training (e.g. gun safety course, tactical firearms training, concealed carry firearm training, NRA training, etc.)
- I have had INFORMAL training.
- I have never had any kind (formal or informal) of firearms training. *Skip to question 13.*

Firearms training

If you have had both formal and informal training, please select only "Formal"

12. **With which type of firearms have you received formal or informal training? (select all that apply)**

Mark only one oval per row.

	Formal	Informal	None
Handgun, revolver			
Handgun, semiautomatic			
Rifle			
Shotgun			
Military-style weapon (e.g. AR15, AK-47, SKS)			
Pellet or airgun			

Firearms handling in the Emergency Department

13. **If you were to encounter a firearm in a patient's possession, how confidently do you feel you could safely handle it until it can safely be turned in to law enforcement?**
Mark only one oval.

- ( ) Extremely confident
- ( ) Moderately confident
- ( ) Somewhat confident
- ( ) Slightly confident
- ( ) Not at all confident

14. **With which steps of safe firearm handling with respect to a revolver-type handgun do you feel proficient? (select all that apply)**
Check all that apply.

- [ ] Treating every firearm as if it is loaded
- [ ] Always pointing the firearm in a safe direction
- [ ] Engaging the safety
- [ ] Unloading the firearm

15. **With which steps of safe firearm handling with respect to a semiautomatic handgun do you feel proficient? (select all that apply)**
Check all that apply.

- [ ] Treating every firearm as if it is loaded
- [ ] Always pointing the firearm in a safe direction
- [ ] Engaging the safety
- [ ] Unloading the firearm

16. **With which steps of safe firearm handling with respect to a rifle do you feel proficient? (select all that apply)**
Check all that apply.

- [ ] Treating every firearm as if it is loaded
- [ ] Always pointing the firearm in a safe direction
- [ ] Engaging the safety
- [ ] Unloading the firearm

17. **With which steps of safe firearm handling with respect to a shotgun do you feel proficient? (select all that apply)**
Check all that apply.

- [ ] Treating every firearm as if it is loaded
- [ ] Always pointing the firearm in a safe direction
- [ ] Engaging the safety
- [ ] Unloading the firearm

18. **With which steps of safe firearm handling with respect to an airgun or pellet gun do you feel proficient? (select all that apply)**
Check all that apply.

- [ ] Treating every firearm as if it is loaded
- [ ] Always pointing the firearm in a safe direction
- [ ] Engaging the safety
- [ ] Unloading the firearm

19. **With which steps of safe firearm handling with respect to a military-style weapon do you feel proficient? (select all that apply)**
Check all that apply.

- [ ] Treating every firearm as if it is loaded
- [ ] Always pointing the firearm in a safe direction
- [ ] Engaging the safety
- [ ] Unloading the firearm

Hospital Safety Policies

20. **Are you aware of a hospital protocol regarding handling and management of firearms discovered in the possession of patients within your primary emergency department?**
Mark only one oval.

- ( ) Yes, my hospital has a protocol regarding handling of a firearm found in a patient’s possession.
- ( ) No, my hospital does not have a protocol regarding handling of a firearm found in a patient’s possession.
- ( ) I am unsure if my hospital has a protocol.

21. **To what extent does your primary emergency department have security personnel present?**
Mark only one oval.

- ( ) 24 hours a day
- ( ) Only on weekdays
- ( ) Only during normal working hours (e.g 8am to 5pm)
- ( ) Only on a call-in basis
- ( ) Never

22. **Do active-duty police officers act as security personnel in your primary emergency department?**
Mark only one oval.

- Yes
- No
- Unsure
- My primary emergency department does not have security personnel.

23. **Do non-police security personnel within your primary emergency department carry firearms while on shift?**
Mark only one oval.

- Yes
- No
- Unsure
- My primary emergency department only employs police as security personnel.
- My primary emergency department does not have security personnel.

Please label each part of the firearm below.

24. **A.**

25. **B.**

26. **C.**

27. **D.**

28. **E.**

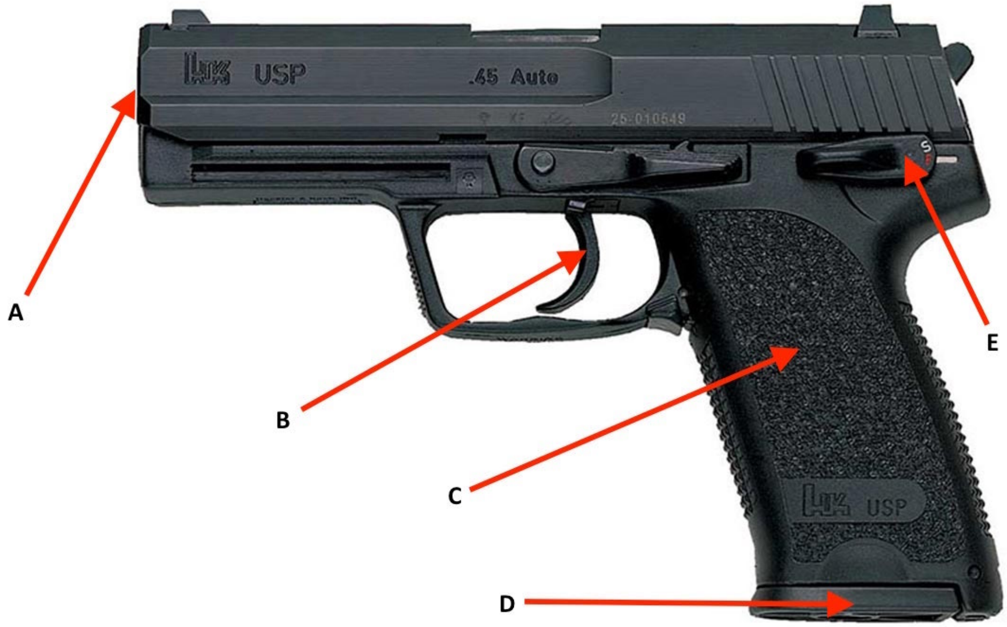

Thank you. Please let us know if you have any questions or concerns.

Powered by
Google Forms
